# Acaricidal and repellent activities of ethanol extracts of nine chinese medicinal herbs against *Rhipicephalus microplus* (Acari: Ixodidae)

**DOI:** 10.1007/s10493-023-00813-3

**Published:** 2023-07-31

**Authors:** Donglinag Li, Shunli Lu, Yichen Jian, Shuqi Cheng, Qianming Zhao, Huizhen Yuan, Nanhao Wang, Yufeng Liu, Sumei Zhang, Longxian Zhang, Rongjun Wang, Fuchun Jian

**Affiliations:** 1 College of Veterinary Medicine, Henan Agricultural University, No.218 Ping’an Avenue, Zhengdong, District, Zhengzhou, Henan 450046 China; 2 College of Animal Science and Technology, Tarim University, Alar, Xinjiang 843300 China

**Keywords:** *Rhipicephalus microplus*, Chinese herbal medicine, Repellent, Acaricide, Ethanol extracts

## Abstract

*Rhipicephalus microplus* is a major threat to the cattle industry worldwide. The intensive use of acaricides and repellents has resulted in drug resistance. Hence, effective and eco-friendly pest control alternatives are urgently needed, especially from natural plant resources. In this study, the acaricidal and repellent activities of nine herbs against the larvae and eggs of *R. microplus* were evaluated. The results showed that ethanol extracts of star anise (*Illicium verum*), chaulmoogra (*Hydnocarpus anthelmintica*), motherwart (*Leonurus artemisia*), mandarin orange peel (citri reticulatae pericarpium, i.e., peel of *Citrus reticulata* fruit), and stemona (*Stemona sessilifolia*) had good contact acaricidal activities of 100, 98, 94, 88 and 86%, respectively, whereas star anise and clove (*Syzygium aromaticum*) had good fumigant acaricidal activities of 98 and 96%, respectively. The hatching inhibition rate of star anise against *R. microplus* eggs was 100%. All nine herbs had good real-time repellent rates, but only castor bean and star anise had repellent effects after 48 h (81.3 and 79.6%, respectively). This is the first report of the acaricidal and repellent activities of these medicinal herbs against *R. microplus*. Ethanol extracts of these herbs might be considered as potential alternatives to chemical acaricides for control of *R*. *microplus*.

## Introduction

Ticks are ectoparasitic arthropods that feed on the blood of birds, reptiles, and mammals, thereby posing serious threats to animal husbandry (Cutler et al. 2021). *Rhipicephalus microplus* is considered a major threat to the cattle industry, accounting for economic losses of approximately 30 billion US dollar annually worldwide, primarily due to decreased quality of meat, milk, and leather products (Gomes and Neves 2018; Estrada-Peña et al. 2006; Grisi et al. 2014). *Rhipicephalus microplus* causes anemia, slows growth, and can spread the parasitic protozoans *Babesia bovis* (causing piroplasmosis), *Babesia bigemina* (causing babesiosis), and the obligate intracellular bacterium *Anaplasma marginale* (causing bovine anaplasmosis), resulting in increased morbidity and mortality of cattle (De Clercq et al. 2012; Pascoeti et al. 2016). Although typically an ectoparasite of cattle, *R. microplus* occasionally infests dogs, sheep, horses, wild animals, and even humans (Esser et al. 2016; McCoy et al. 2013; Rodríguez-Vivas et al. 2016).

At present, conventional synthetic acaricides, such as organophosphates, pyrethroids, amidines, macrocyclic lactones, benzoylphenylureas, and phenylpyrazoles, are used for eradication and control of ticks (Jain et al. 2021; Adenubi et al. 2018). However, the repeated use of these compounds often results in the development of acaricide resistance, accumulation of chemical residues in food, and adverse environmental impacts (Baran et al. 2020; Lunguinho et al. 2021). Hence, effective and eco-friendly pest control alternatives are urgently needed.

Bioactive plants for control of ticks offer several advantages, such as low toxicity to non-target organisms, short environmental persistence, and biodegradation to nontoxic products (Baran et al. 2020; Fetoh and Asiry 2012; Ahmed et al. 2020). Recent studies have focused on natural substances, such as secondary plant metabolites with acaricidal or repellent activities, to protect livestock against ticks (Lunguinho et al. 2021). Notably, essential oils derived from clove, cottonseed, and lemon grass have been investigated as potential substitutes for synthetic pesticides (Valente et al. 2017; Jain et al. 2020; Apel et al. 2009; Castro et al. 2018; Santos and Vogel 2012). However, few studies have evaluated ethanol extracts as potential acaricides and repellents against ticks. Therefore, the aim of the present study was to investigate the safety and effectiveness of crude ethanol extracts of nine plants (mandarin orange peel, star anise, motherwort, clove, chaulmoogra tree, stemona, castor bean, shrubby sophora, and box bean) as alternative acaricides and repellents for the management of *R*. *microplus*.

## Materials and methods

### Herbs and chemicals

Clove (*Syzygium aromaticum*) and star anise (*Illicium verum*) were purchased from Beijing Tong Ren Tang (Beijing, China), shrubby sophora (*Sophora flavescens*) and motherwort (*Leonurus cardiac*) from Henan Zhangzhongjing Pharmacy (Zhengzhou, China), and mandarin orange peel (Citri reticulatae pericarpium, CRP; orange-colored *Citrus reticulata* Blanco fruit peel, ‘chenpi’ in Chinese), chaulmoogra (*Hydnocarpus anthelmintica*), stemona (*Stemona sessilifolia*), castor bean (*Ricinus communis*), and box bean (*Entada phaseoloides*) from online Wenzexuan Traditional Chinese Medicine Shop (Hangzhou, China). As a positive control, 100 mg/mL ivermectin was obtained from Henan Anjin Biological Technology (Xinxiang, China). Anhydrous ethanol (analytical grade) was acquired from Tianjin Fuyu Fine Chemical (Tianjin, China). As a negative control, 0.9% sodium chloride was purchased from Henan Kelun Pharmaceutical (Anyang, China).

### Preparation of herbal material and extraction

Extractions of the active herbal ingredients were conducted as reported by Jian et al. (2022a). Briefly, 50 g of each herb were ground, soaked in 200 mL of 90% ethanol for 1 week, then filtered through gauze and mixed in 100 mL of 90% ethanol. After 24 h, the mixture was filtered and the supernatant was collected, then the two filtrates were combined and centrifuged at 3000× ***g*** for 10 min, then heated to evaporate the ethanol and concentrated into a paste, which was dissolved in 50 mL of 0.9% NaCl. Finally, the extracted liquid was diluted with 0.9% NaCl to concentrations of 0.1, 0.325, 0.55 or 0.775 g/mL, which were stored at 4 ℃ until further use.

### Collection and identification of ticks

Engorged female ticks were collected from local herds in Jiyuan City, Henan Province, China, stored in 2-mL centrifuge tubes containing wet cotton balls, and transported to the Parasitology Laboratory of Henan Agricultural University (Zhengzhou, China) for identification (Black et al., 1994). Ticks confirmed as *R. microplus* were transferred to 60-mm culture dishes and stored at a constant 28 ℃ and 90% relative humidity to promote spawning and hatching. Larvae were stored at 4 ℃ until further use in the subsequent experiments.

### Ovicidal activities of the ethanol extracts

Ovicidal tests were performed with reference to ‘Pesticides guidelines for laboratory bioactivity tests. Part 5: The dipping test for insecticide ovicidal activity’ (www.chinanyrule.com). *Rhipicephalus microplus* eggs (n = 10) in good condition were attached to white cardboard (15 × 25 mm) with double-sided tape, immersed in herbal extract for 1 min, removed, blotted dry, and transferred to a Petri dish without the herbal extract. Each sample was assayed in triplicate. NaCl solution was used as a blank control and 100 mg/mL ivermectin as a positive control. Treated eggs were incubated at a constant 28 °C and 90% RH. Eggs were observed for hatching every 24 h until the hatching rate of the blank control was > 80%, and then continuously observed for an additional 48 h.

### Acaricidal activities of the ethanol extracts

#### Fumigation

The fumigation method was performed with reference to Jian et al. (2022a). Cotton balls of equal sizes were evenly saturated with 0.5 mL of each herbal extract (0.1, 0.325, 0.55, 0.775 or 1 g/mL) and then dried. *Rhipicephalus microplus* larvae (n = 10) of uniform size were transferred into 2-mL centrifuge tubes. Upon observation of normal movements of ticks, the prepared cotton balls were added to the centrifuge tube and removed after 1 h. All treatments were repeated 5×. Distilled water was used as a blank control, 0.9% NaCl solution as a negative control, and ivermectin as a positive control. Treated ticks were incubated at a constant 28 °C and 90% RH. The mortality rate was recorded after 48 h. Larvae were considered dead if there was no response after continuous stimulation with a needle for 1 min.

#### Impregnated filter paper method

A filter paper was placed on the bottom of a Petri dish (60 × 15 mm) and 1 mL of the herbal extract (0.1, 0.325, 0.55, 0.775 or 1 g/mL) was evenly dispersed, then exposed to air to dry naturally for 24 h. *Rhipicephalus microplus* larvae (n = 10), crawling normally, with limbs intact and of uniform size, were transferred to the Petri dishes containing herbal extract. After 1 h, the larvae were transferred to clean Petri dishes. Each treatment was replicated 5×. Distilled water was used as a blank control, 0.9% NaCl solution as a negative control, and 100 mg/mL ivermectin as a positive control. Petri dishes were incubated at a constant 28 °C and 90% RH. After 48 h, the mortality rate was recorded. Larvae were considered dead if there was no response after continuous stimulation with a needle for 1 min.

#### Dip method

*Rhipicephalus microplus* larvae (n = 10) – crawling normally, limbs intact and uniform size – were transferred to a Petri dish containing 5 mL of the herbal extract (0.1, 0.325, 0.55, 0.775 or 1 g/mL). After being immersed for 1 min, larvae were transferred to a clean Petri dish. Each treatment was replicated 5×. Distilled water was used as a blank control, 0.9% NaCl solution as a negative control, and 100 mg/mL ivermectin as a positive control. The Petri dishes were incubated at a constant 28 °C and 90% RH. After 48 h, the mortality rate was recorded. Larvae were considered dead if there was no response after continuous stimulation with a needle for 1 min.

### Repellent activities of ethanol extracts

**Fig. 1:**
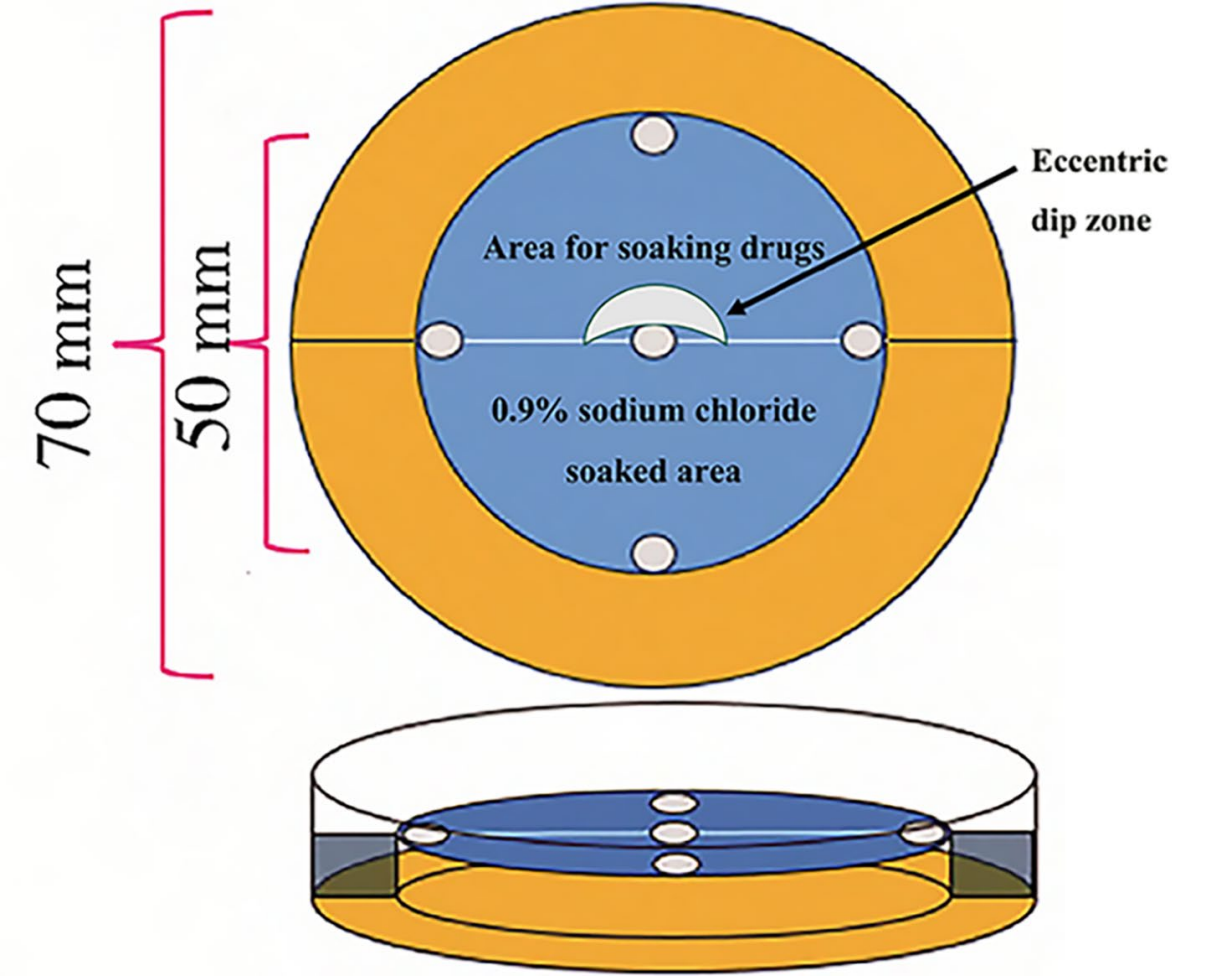
Larval tick repellent device

Filter papers (5 cm diameter) were cut into two semicircles, which were soaked in the ethanol extracts (0.1, 0.325, 0.55, 0.775 or 1 g/mL) and 0.9% NaCl solution for 10 min, respectively, then exposed to air to dry naturally for 24 h, recombined, and placed in a larval tick repellent device (Zhao et al. 2022; Fig. 1). For each experiment, 20 larvae were placed at the circular ‘eccentric dip zone’ with free movement on the field. Distribution of the larvae on the filter paper was observed after 5 min of light avoidance, and the repellent rate was calculated. Each experiment was performed in triplicate with different larvae. Then, the filter paper was exposed to air at room temperature (25–28 °C) and tested again after 48 h. The duration of the repellent effect of ethanol extract against the larvae was determined.

### Determination of chinese herbal medicine ethanol extract composition

Based on the results of the ‘Impregnated filter paper’ method, alcoholic extracts of the herbs with the best acaricidal activity (star anise and chaulmoogra) were selected and sent to Biomarker Technologies (Beiing, China) for compositional analysis. Based on the UHPLC-QE Orbitrap platform, the qualitative and quantitative compositional analysis of two ethanolic extract samples of the herb was performed. LC-MS/MS analyses were performed using an UHPLC system (1290, Agilent Technologies) with a UPLC HSS T3 column (1.8 μm 2.1 × 100 mm, Waters) coupled to Q Exactive (Orbitrap MS, Thermo).The mobile phase consisted of positive: 0.1% formic acid in water, and negative :5 mM ammonium acetate in water (A) and acetonitrile (B), carried with elution gradient as follows: 0 min, 1% B; 1 min, 1% B; 8 min, 99% B; 10 min, 99% B; 10.1 min, 1% B; 12 min, 1% B, which was delivered at 0.5 mL min^− 1^. The injection volume was 1 µL. The QE mass spectrometer was used for its ability to acquire MS/MS spectra on aninformation-dependent basis (IDA) during an LC/MS experiment. In this mode, the acquisition software (Xcalibur v.4.0.27, Thermo) continuously evaluates the full scan survey MS data as it collects and triggers the acquisition of MS/MS spectra depending on preselected criteria. ESI source conditions were set as follows: sheath gas flow rate as 45 Arb, aux gas flow rate as 15 Arb, capillary temperature 320 ℃, full ms resolution as 70,000, MS/MS resolution as 17,500, collision energy as 20/40/60 eV in NCE model, ion spray voltage floating (ISVF) 3.8 or -3.1 kV in positive or negative modes, respectively. Identification of compounds was based on a comparison of mass spectra of each peak with those of authentic samples in a mass spectrum library. The percentages of compounds were calculated by the area normalization method.

### Statistical analysis

The mortality rate (%) was calculated as [number of dead larvae / total number of larvae] × 100%. In addition, the corrected mortality rate (%) was calculated as [(mortality − mortality of blank control group) / (1 − mortality of blank control group)] × 100% (Jian et al., 2022a). If the blank control mortality rate was < 5%, no correction was required. Hatching inhibition rate (%) was calculated as [number of unhatched eggs / total number of treated eggs] × 100% and the corrected hatching inhibition rate (%) as [(hatching inhibition rate − blank control group hatching inhibition rate) / (1 − blank control group hatching inhibition rate)] × 100%. If the blank control hatching inhibition rate was < 5%, no correction was required. The repellent rate (%) was calculated as [(number of insects in the control area − number of insects in the treated area) / number of insects in the control area] × 100% (Zhao et al. 2022).

Mean (and SE) mortality rate, egg hatching inhibition rate, and repellent rate were calculated with IBM SPSS Statistics for Windows v.26.0 (IBM Corporation, Armonk, NY, USA), analyzed with ANOVA and compared using least significant difference (LSD) tests (α = 0.05). The median lethal concentration (LC_50_) and median repellent concentration (RC_50_) were calculated with the Probit algorithm. The coefficient of determination (R^2^) and regression equation were calculated by linear regression. Graphs were generated with GraphPad Prism v.8.0.2 (GraphPad Software, San Diego, CA, USA).

## Results

### Ovicidal activity of the ethanol extracts

At 1 g/mL, ethanol extracts of three herbs inhibited egg hatching, showing good egg hatching inhibitory activity: star anise (100%), stemona (66.7%) and CRP (60%). For comparison, the rates of the positive, negative, and blank controls were 83.3, 3.3 and 0%, respectively (Table 1).

**Table 1:** Mean (± SE) ovicidal and acaricidal activities of ethanol extracts of nine herbs and three controls against *Rhipicephalus microplus*

Treatment*	Adjusted hatching inhibition rate (%)	Adjusted mortality after 48 h (%)		
		Fumigation	Impregnated filter paper method	Dip method
Star anise	100a	98 ± 2a	100a	17.5 ± 10.9d
Shrubby sophora	10 ± 5.8fg	0b	16 ± 6.8bc	0d
CRP	60 ± 17.3bcd	2 ± 2b	88 ± 7.4d	66.4 ± 11.5b
Castor bean	40 ± 11.6cde	0b	14 ± 7.5 cd	2.2 ± 2.2d
Clove	36.7 ± 14.5def	96 ± 4a	28.9 ± 4.5b	0d
Box bean	10fg	0b	26 ± 7.5bc	2.5 ± 2.5d
Chaulmoogra	13.3 ± 6.7efg	0b	98 ± 2a	92.8 ± 3a
Stemona	66.7 ± 8.8bc	0b	86 ± 5.1a	12.2 ± 5.6d
Motherwort	13.3 ± 6.7efg	0b	94 ± 4a	43.9 ± 9.9c
Negative control	3.3 ± 3.3g	0b	0d	0d
Positive control	83.3 ± 12ab	100a	100a	97.8 ± 2.2a
Blank control	0g	0b	0d	0d

Negative control: 0.9% NaCl solution; positive control: 100 mg/mL ivermectin; blank control: distilled water. CRP: citri reticulatae pericarpium. Means within a column followed by different letters are significantly different (LSD: *P* < 0.05)

*Test concentration of alcohol extracts of Chinese herbal medicines was 1.0 g/mL

### Acaricidal activities of the ethanol extracts

**Fig. 2:**
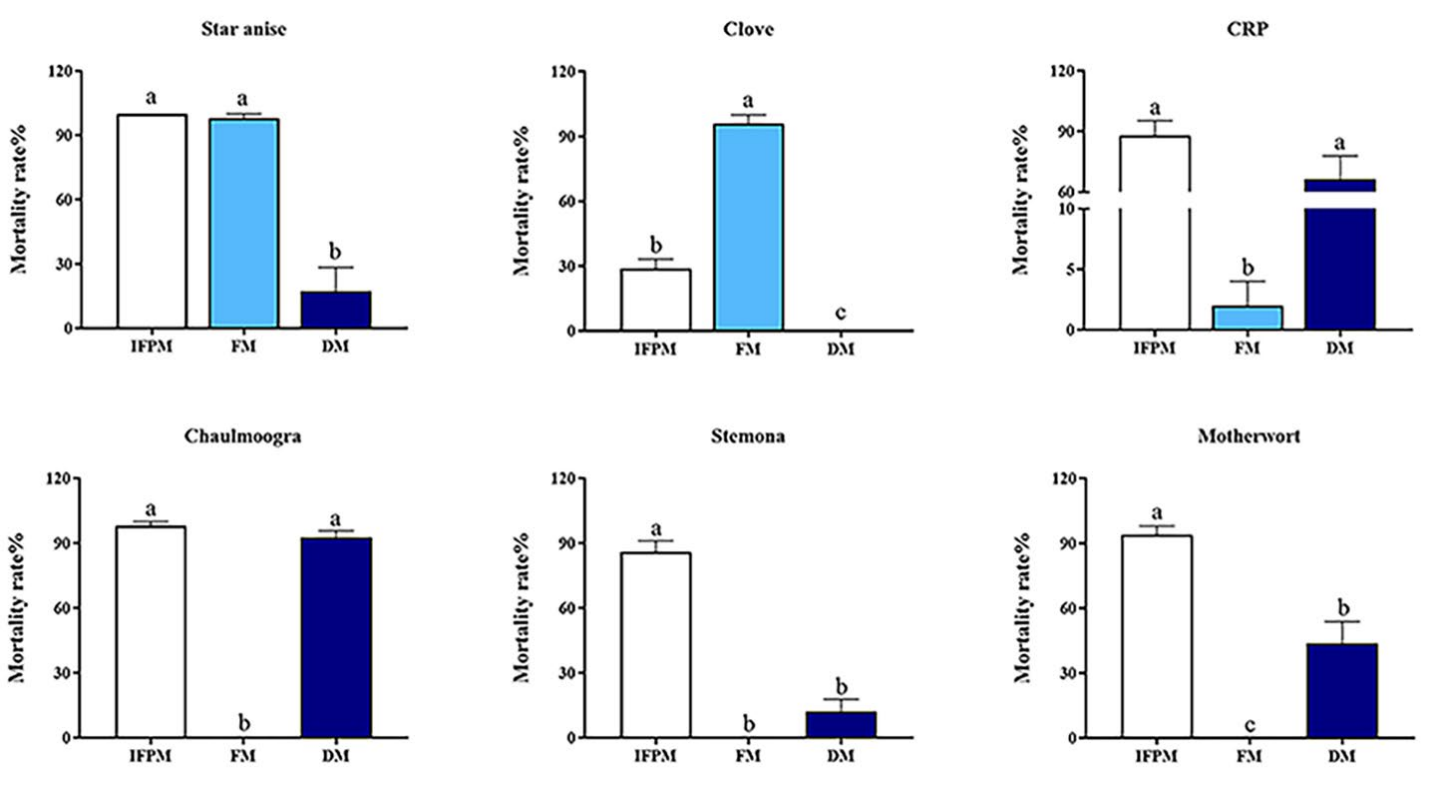
Mean (+ SE) overall acaricidal activities (% adjusted mortality after 48 h) of three methods against *Rhipicephalus microplus* larvae: impregnated filter paper method (IFPM), fumigation (FM), and dip method (DM). CRP = citri reticulatae pericarpium. Means within a panel capped with different letters are significantly different (LSD: *P* < 0.05)

The delivery method significantly influenced the acaricidal activity of ethanol extracts against *R. microplus* larvae. As shown in Fig. 2, the highest acaricidal activity with the impregnated filter paper method was by star anise (100%) > chaulmoogra (98%) > motherwort (94%) > CRP (88%) > stemona (86%), whereas clove (96%) had the highest acaricidal activity with the fumigation method, and chaulmoogra (92.8%) had the highest acaricidal activity with the dip method.

#### Fumigation

**Fig. 3:**
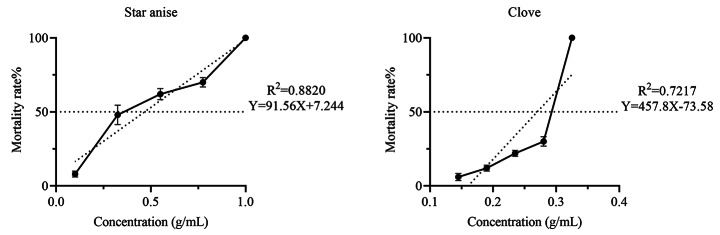
Linear regression analysis of the acaricidal activities (mean ± SE % mortality) of ethanol extracts of star anise and clove against *Rhipicephalus microplus* larvae (fumigation assay)

At 1 g/mL, ethanol extract of star anise had the highest acaricidal rate (98%) followed by clove (96%), whereas the other seven herbs had no acaricidal activity. For comparison, the acaricidal rates of the positive, negative, and blank controls were 100, 0, and 0%, respectively (Table 1). The LC_50_ and LC_90_ of star anise are 0.457 and 0.884 g/mL, respectively (Table 2; Fig. 3).

**Table 2:** Probit regression analysis of the acaricidal activities of ethanol extracts of various herbs against *Rhipicephalus microplus*larvae

Test method	Treatment	LC_50_ (g/mL)	95% confidence interval	LC_90_ (g/mL)	95% CI	Pearson χ^2^
Fumigation	Star anise	0.457	0.285–0.601	0.884	0.712–1.314	2.855
Impregnated filter paper method	Star anise	0.367	0.086–0.537	0.912	0.701–1.572	1.563
	CPR	0.312	-0.007-0.476	0.836	0.640–1.430	1.790
	Chaulmoogra	0.204	0.173–0.229	0.274	0.246–0.340	1.239
	Stemona	0.648	0.469–0.892	1.208	0.942–2.071	0.641
	Motherwort	0.247	0.052–0.364	0.549	0.422–0.879	1.193

CRP: citri reticulatae pericarpium

#### Impregnated filter paper method

**Fig. 4:**
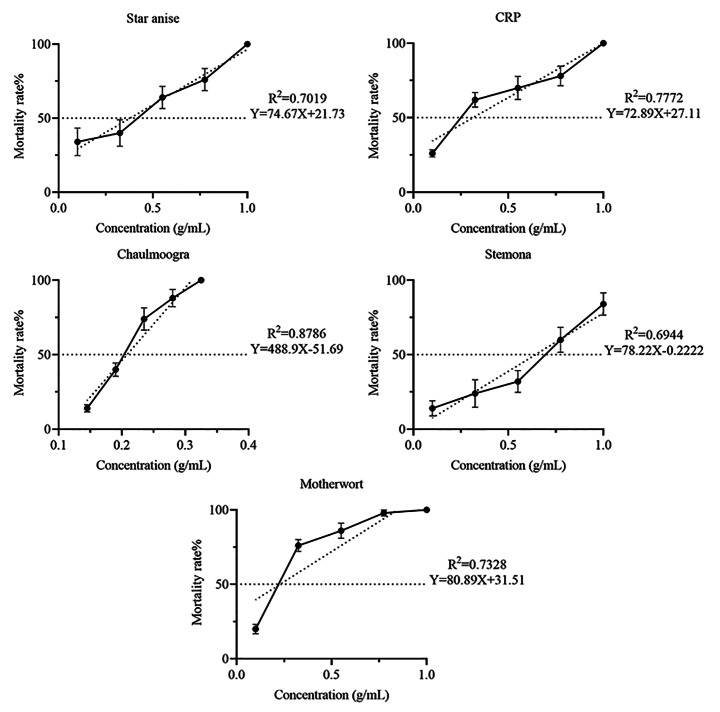
Linear regression analysis of the acaricidal activities (mean ± SE % mortality) of ethanol extracts of star anise, chaulmoogra, CRP (= citri reticulatae pericarpium), motherwort, and stemona against *Rhipicephalus microplus* larvae (impregnated filter paper method)

At 1 g/mL, ethanol extracts of five herbs had acaricidal rates of > 80%, where star anise had the highest corrected mortality rate of 100%, followed by chaulmoogra (98%), motherwort (94%), CRP (88%), and stemona (86%). For comparison, the acaricidal rates of the positive, negative, and blank controls were 100, 0, and 0%, respectively (Table 1). In terms of LC_50_ and LC_90_, chaulmoogra (LC_50_ and LC_90_ = 0.058 and 0.408 g/mL, respectively) was the most effective, followed by motherwort (0.247 and 0.549), CRP (0.312 and 0.836), star anise (0.367 and 0.912), and stemona (0.648 and 1.208) (Table 2; Fig. 4).

#### Dip method

At 1 g/mL, ethanol extracts of only two herbs had an acaricidal rate of > 50%, where chaulmoogra (92.8%) > CRP (66.4%) > motherwort (43.9%). The acaricidal rates of the other six ethanol extracts were < 20%. For comparison, the acaricidal rates of the positive, negative, and blank controls were 97.8, 0, and 0%, respectively (Table 1).

### Repellent activities of the ethanol extracts

**Fig. 5:**
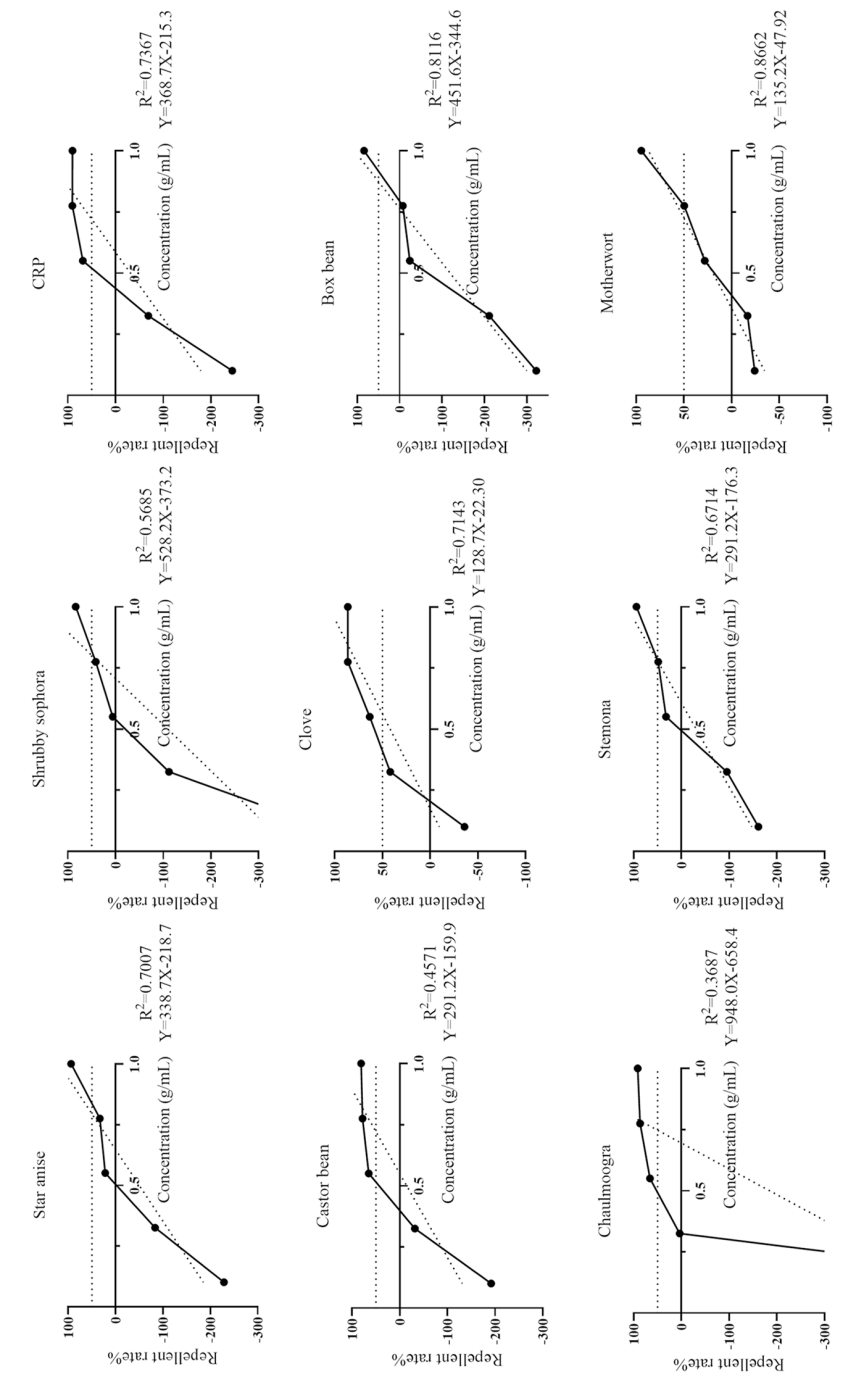
Linear regression analysis of the repellent activities of the ethanol extracts of nine herbs against *Rhipicephalus microplus* larvae. CRP = citri reticulatae pericarpium

At 1 g/mL, ethanolic extracts of all nine herbs showed high repellency at 0 h (all > 80%; Table 3) and significantly different from that of the negative control (*p* < 0.01). Notably, repellent rates of the ethanol extracts of all nine herbs decreased with time, but to different degrees. After 48 h, only castor bean (81.3%), star anise (79.6%), chaulmoogra (66.3%), and motherwort (66.3%) maintained repellency rates > 50%. As shown in Fig. 5, the repellent rates of the ethanol extracts of all nine herbs gradually increased with concentrations of 0–1 g/mL, with maximum rates at 1 g/mL. There was a linear relationship between the repellency rate and concentration. The ethanol extract of clove had the highest repellent activity (RC_50_ = 0.562 g/mL), whereas the RC_50_ values of the ethanol extract of the other eight herbs were > 0.7 g/mL.

**Table 3:** Mean (± SE) repellent activities of ethanol extracts of nine herbs and two controls against *Rhipicephalus microplus* larvae

Treatment^*^	Repellent rate after 0 h (%)	Repellent rate after 48 h (%)	RC_50_ (0 h)
Star anise	94.12 ± 5.88a	79.63 ± 4.63a	0.793
Shrubby sophora	83.96 ± 13.50a	33.33 ± 19.25a	0.801
CRP	90.61 ± 4.13a	-156.84 ± 157.38b	0.720
Castor bean	94.54 ± 8.12a	81.25 ± 3.21a	0.721
Clove	86.48 ± 4.13a	26.49 ± 13.75a	0.562
Box bean	84.03 ± 5.76a	11.11 ± 11.11a	0.874
Chaulmoogra	91.67 ± 8.33a	66.27 ± 5.16a	0.747
Stemona	94.54 ± 3.21a	26.49 ± 13.75a	0.777
Motherwort	94.74 ± 0.00a	66.27 ± 5.16a	0.724
Negative control	-123.81 ± 38.10b	-161.11 ± 73.49b	/
Positive control	100a	100a	/

Negative control: 0.9% NaCl solution; positive control: 100 mg/mL ivermectin; blank control group: distilled water. CRP: Citri reticulatae pericarpium. RC_50_ (0 h) represents 50% repellent quantity of Chinese herbal medicine to larvae of *R. microplus* at 0 h

Means within a column followed by different letters are significantly different (LSD: *P* < 0.05)

*Test concentration of alcohol extracts of Chinese herbal medicines was 1.0 g/mL

### Main components of ethanol extract of star anise and chaulmoogra

The main chemical composition of ethanol extract of star anise and chaulmoogra is presented in Table 4. LC-MS/MS analyses showed that in total 892 metabolites were detected. Among them, the main compounds of star anise are phenethylacetate (7.8%), 4-hydroxybenzaldehyd (4.2%) and isosafrole (3.6%). The main compounds of chaulmoogra are tuberostemonine (5.6%) and vanillyl alcohol (5.4%).

**Table 4:** The main compounds of star anise and chaulmoogra

Star anise				Chaulmoogra		
Compounds	Relative abundance	%		Compounds	Relative abundance	%
Phenethylacetate	5,094,536,006	7.8		Tuberostemonine	3,111,484,605	5.6
4-Hydroxybenzaldehyde	2,764,332,275	4.2		Vanillyl alcohol	2,974,508,553	5.4
Isosafrole	2,358,961,700	3.6		Choline [M]+	2,917,888,998	5.3
Quercetin-3-O-xyloside	2,073,357,887	3.2		4-Trimethylammoniobutanoic acid	1,641,589,648	3.0
Cis-Aconitic acid	1,879,215,246	2.9		D-Gluconic acid	1,379,346,714	2.5
β-Thujaplicin	1,567,653,491	2.4		α, β-Thujaplicin	1,372,047,170	2.5
Choline [M]+	1,355,462,385	2.1		Cafestol	1,227,630,594	2.2
N-Fructosyl pyroglutamate	1,203,082,390	1.8		Methyl hexadecanoate	1,118,073,016	2.0
Quercetin-3-O-galactoside	1,042,928,036	1.6		2-Chloro-DL-Phenylalanine	1,108,018,911	2.0
Benzylidenacetone	1,020,821,503	1.6		Malic acid	1,104,902,096	2.0

## Discussion

Many recent studies have reported anti-mite and anti-tick activities of various herbs. In the present study, several of the ethanol extracts showed good acaricidal activity, namely star anise (100%), chaulmoogra (98%), motherwort (94%), CRP (88%), and stemona (86%). This study is the first to report acaricidal activities of ethanol extracts of star anise, chaulmoogra, motherwort, and CRP against *R*. *microplus*. Notably, the acaricidal activity of the ethanol extract of stemona was significantly higher in the present study than the one reported by Kongkiatpaiboon et al. (2014) in an in vitro acaricidal test.

Star anise has been reported as a broad-spectrum insecticide primarily because of the presence of trans-anethole, which can be used directly as an insecticide or synergistically as an adjunct to other insecticides (Park et al. 2016). Jian et al. (2022b) reported that the contact mortality of the ethanol extract of star anise was 96% against the adult chicken mite (*Dermanyssus gallinae*) with a LD_50_ of 0.159 g/mL. Star anise essential oil has a relatively high content of trans-anisidine and, thus, better insecticidal activities against the Indian meal moth (*Plodia interpunctella*) and the litter beetle (*Alphitobius diaperinus*) with insecticidal rates of > 90% at lower doses (Choi et al. 2022; Peter et al. 2022). In this study, star anise had significant acaricidal effects against *R. microplus* larvae by the fumigation method (98%), impregnated filter paper method (100%), and ovicidal test (100%).

In traditional Chinese medicine, chaulmoogra has the effect of ‘dispelling wind’ and ‘drying dampness’, and is mainly used for treatment of leprosy, skin diseases, and worm infection (Zou et al. 2017). The main components of the chaulmoogra seed are fatty acid triglycerides, sterols, flavonoids, and flavonoid lignans. The seed oil is commonly used for medicinal purposes, owing to the highest content of gigantic acid (Sahoo et al. 2014), and as an acaricide against mites. A study by Song et al. (2002) found good acaricidal activities of a 95% ethanol extract of chaulmoogra against the mite *Sarcoptes scabiei*, which causes scabies of rabbits, and mites of the *Psoroptes* genus, which cause mange in domesticated and wild ungulates. Yang et al. (2013) reported that a water extract of chaulmoogra was effective against *S*. *scabiei* var. canis, the mite that causes canine acariasis.

To date, more than 300 chemical components have been isolated from stemona, including the alkaloid monomers stemofoline and stemospironine with proven insecticidal effects (Chalom et al. 2021). Both stemofoline and stemospironine are toxic to silkworm larvae, but employ different mechanisms (Sakata et al. 1978). The insecticidal mechanism of stemona mainly includes inhibition of acetylcholinesterase activity (Lai et al. 2013) as a toxicant against the acetylcholine receptor (Tang et al. 2008). Stemona has good insecticidal activities against a range of insects, but yet is harmless to humans. Thus, stemona is widely used in clinical practice and in the field of animal husbandry (Zhu et al. 2021). Mungkornasawakul et al. (2004) found that the alkaloids stemocurtisine (LC_50_ = 18 ppm), stemocurtisinol (LC_50_ = 39 ppm), and oxyprotostemonine (LC_50_ = 4 ppm) from the roots of *Stemona curtisii* had good larvicidal activity against the mosquito *Anopheles minimus*, which is the primary vector of malaria in India. Brem et al. (2002) also demonstrated significant repellent and insecticidal effects of stemofoline.

Motherwort is commonly used for treatment of epilepsy, menstrual disorders, arterial diseases, and gastrointestinal disorders. The antioxidant and anti-inflammatory effects of motherwort and CRP have been linked to various flavonoids, terpenes, and alkaloids (Koshovyi et al. 2021). However, relatively few studies have investigated the insecticidal activities of motherwort and CRP. Previous studies by Jian et al. (2022a, b) found good acaricidal activity of ethanol extracts of motherwort against the northern fowl mite (*Ornithonyssus sylviarum*) and *D. gallinae*. The main active ingredients of CRP include volatile oils and the citrus flavonoids hesperidin, neohesperidin, and naringenin that not only act alone, but also synergistically with other drugs (Yu et al. 2018; Xu et al., 2012). Bordin et al. (2021) reported that essential oils of *Citrus* spp. have low acaricidal effects against *D. gallinae*, whereas Peniche-Cardeña et al. (2022) found that the *n*-hexane fraction of *Citrus paradisi* with the *n*-hexane and dichloromethane fractions of lychee (*Litchi chinensis*) had a synergistic acaricidal effect against *R. microplus.*

With the fumigation method, the ethanol extract of clove (96%) showed high acaricidal activity against *R. microplus* larvae, possibly due to the high content of volatile oils composed of eugenol (78–95%), acetyl eugenol (7.3%), and ß-caryophyllene (9%). At 3 µg/m^2^, the ethanol extract of clove was 100% effective against *D. gallinae* (Lee et al. 2019; Tabari et al. 2020). Moreover, the greater toxicity of the ethanol extract of clove with the fumigation method might be due to the faster rate that vapor passes through the respiratory tract as compared to the tactile method (Ribeiro et al. 2019).

With the dip method, only chaulmoogra (92.8%) demonstrated good acaricidal activity, probably because of the relatively short contact time between the ethanol extract of most of the tested herbs and the tick larvae. In contrast, with the impregnated filter paper method, the herbal extract was distributed uniformly, which increased the contact time, demonstrating that the impregnated filter paper method is more suitable for the tactile method.

In a review by Nwanade et al. (2020), a survey summarizing articles on botanical acaricides and repellents from 2017 to 2019, it was found that not all species and life stages of ticks were suitable for every screening method. The larval packet test (LPT) was the most preferred in evaluating larvicidal activity. Also, most plants showed good larvicidal and adulticidal activities against ticks. But the age of ticks might cause a differential response to acaricides and repellents. Further observations revealed that the larval stage was more susceptible (Adenubi et al. 2018). Essential oils (EOs) of *Cinnamomum cassia* and (*E*)-cinnamaldehyde exhibited acaricidal activity, with LC_50_ values of 3.81 and 3.15 mg/mL, respectively, against the larvae of *Haemaphysalis longicornis*, and 21.31 and 16.93 mg/mL, respectively, against *H. longicornis* nymphs (Nwanade et al. 2021). Differences in the efficacy may be attributed to the relative concentration of the various functional compounds and their mode of action on the various life stages (Nwanade et al. 2020).

The results of the ovicidal test showed that the ovicidal activity of star anise, stemona, and CRP exceeded 50%, but only star anise reached 100%, whereas the positive control was only 83.3%, possibly due to the difficulty of some chemicals to penetrate the eggs or the time of exposure. Li et al. (2020, 2021) exposed *S. scabiei* eggs to 25% benzyl benzoate for up to 12 h, whereas the classical technique with a filter paper results in an exposure duration of up to 5 days. With a 12 h-exposure period, 19.3% of eggs were able to hatch, whereas only 8.3% of eggs finally hatched with the filter paper method.

Currently, three methods are generally employed by the livestock industry to control ticks: environmental spraying, medicated baths, and injectables. Although these methods can effectively kill parasitic ticks, the ticks may have already bitten the host and transmitted pathogens. In addition, the overuse of chemical insecticides can promote drug resistance and cause side effects in non-target species, which could impact the ecosystem and human health (Ferreira et al. 2017; Jordan et al. 2012). The mechanisms of action of repellents mainly involve odors that ticks avoid. In addition, relatively small amounts of repellents are sufficient because of relatively high volatility, thereby avoiding resistance (Zhao et al. 2021).

In this study, all nine herbs had a real-time (i.e., the filter paper dries naturally for 24 h, and is then immediately subjected to a repellent test) repellency rate of > 80%, but the ethanol extracts of only four herbs had a repellency rate of > 50% after 48 h, which included castor bean (81.3%) and star anise (79.6%), possibly due to the high volatility of these major constituents (Pålsson et al. 2008). Their study that evaluated the repellent effect of the EOs of *Tanacetum vulgare* (1,8-cineole, 7.6%) against nymphs of *Ixodes ricinus* also reported the decrease in its long-term repellent properties. Indeed, El-Seedi et al. (2012) suggested that, although strong, the repellent effect of the EOs of *Rosmarinus officinalis* (1,8-cineole, 51.8%) against nymphs of the tick *I. ricinus* decreases over the long term, probably because of the high volatility of 1,8-cineole. It was confirmed that the best insect repellent effect of most of the herbs studied in this study occurred only at the highest concentration and the shortest drying time assessed. Hence, future studies should focus on the main active ingredients and delay the volatilization rate of the active ingredients to increase the repellent time.

Natural products or extracts may be ideal tick control agents since they may be able to reduce the development of resistance and are not harmful to the environment. However, some disadvantages of plant-based products include short duration of activity, the potential for skin sensitization and allergies, and many plant-based compounds are toxic to some animals (Vigan 2010). The drawback in the research for new plant-based tick repellents and acaricides is the lack of a standardized testing method (Adenubi et al. 2018). Different batches of plants, different parts of the plant, different extraction methods and different testing methods may all lead to different results. Future research may be needed to develop standardized testing methods for plant-based insect repellents and acaricides in order to uniformly evaluate the merits of drugs of plant origin. And research on the toxicity of plant-based products could be strengthened to reduce side effects on humans and animals.

## Conclusions

The ethanol extracts of star anise, CRP, chaulmoogra, stemona, and motherwort had strong acaricidal effects against *R. microplus*, whereas the ethanol extracts of star anise and clove also had good fumigant acaricidal effects, and the hatching inhibition rate of *R. microplus* eggs by star anise reached 100%. Therefore, these medicinal Chinese herbs should be further investigated as potential alternative drugs for tick control. In addition, the results of this study showed that all nine herbs had good real-time repellent effects, but only castor bean and star anise had good delayed repellent effects. Therefore, delaying volatilization of the repellent components is an important direction for future repellent development. Hence, further studies are warranted to identify the effective acaricidal components of these herbs, determine the synergistic effects among different components, and establish appropriate delivery methods to develop safer and more effective acaricides and repellents.
